# The Challenges of Mitochondrial Replacement

**DOI:** 10.1371/journal.pgen.1004315

**Published:** 2014-04-24

**Authors:** Patrick F. Chinnery, Lyndsey Craven, Shoukhrat Mitalipov, James B. Stewart, Mary Herbert, Douglass M. Turnbull

**Affiliations:** 1 Wellcome Trust Centre for Mitochondrial Research, Institute for Ageing and Health, Newcastle University, Newcastle upon Tyne, United Kingdom; 2 Institute of Genetic Medicine, Newcastle University, Central Parkway, Newcastle upon Tyne, United Kingdom; 3 Division of Reproductive & Developmental Sciences, Oregon National Primate Research Center, Oregon Health & Science University, Beaverton, Oregon, United States of America; 4 Max Planck Institute for Biology of Ageing, Cologne, Germany; 5 Newcastle Fertility Centre, International Centre for Life, Newcastle University, Newcastle upon Tyne, United Kingdom; Royal Children's Hospital, Australia

New advances in medicine often raise challenges, and none more so than those involving the manipulation of human oocytes and embryos. Issues around clinical need and ethical considerations must be taken into account, as well as the safety of the proposed technique. The discussion around the proposed mitochondrial replacement techniques to prevent the transmission of mitochondrial DNA disease has perhaps raised more challenges than most [1].

Mitochondrial DNA diseases are both common and, in their severest forms, devastating [2]. There are limited treatments available for these patients, and those that are successful are focused on treating complications such as epilepsy and cardiac disease [3]. Mitochondrial DNA diseases are transmitted maternally, and for families carrying these mutations, a major, and justifiable, desire is to have unaffected children. For some women, preimplantation or prenatal diagnosis may be helpful [4], [5], but for other women, these techniques will not result in disease-free offspring and the only options available are either oocyte donation or mitochondrial replacement at the oocyte or zygote stage. The need for this technique for these families is well established, as are the experimental methods that are required for mitochondrial replacement [6]–[8]. The major scientific concerns for those of us working in the field revolve around safety and efficacy.

In the United Kingdom, the Human Fertilisation and Embryology Authority (HFEA) recently considered the safety issues after extensive expert and public consultation [9]. This independent group of scientists reviewed all the evidence and concluded that mitochondrial replacement techniques have the potential to be used for patients with mitochondrial DNA disease, although further experiments are required before introduction into clinical practice, to provide further reassurance with respect to efficiency and safety. Recently [10], it has been suggested that the possibility of a harmful interaction between the mitochondrial and nuclear genomes has not been given due weight. Should we therefore stop further clinical developments in this area with immediate effect?

The authors raise an interesting evolutionary argument that the human mitochondrial genome co-evolves with the nuclear genome in females, raising the possibility of a conflict with the paternal nuclear genome. They suggest Leber's hereditary optic neuropathy (LHON) and male infertility could be potential examples of this in humans [10]. Firstly, LHON is not a male-limited disease as they suggest [11]. The disorder affects ∼10% of women carrying specific mtDNA mutations, and although there is increased penetrance in males, strenuous efforts have failed to identify a nuclear modifier gene to date, and the increased penetrance in men could simply reflect the absence of oestrogens [12]. As regards male infertility, there is no convincing evidence in man that inherited variants of mtDNA are at all relevant in the general population [13], [14]. Indeed it is interesting that even in male patients with pathogenic mitochondrial DNA mutations, such as LHON, reduced fertility has not been reported to be a major clinical feature.

The studies in macaques are also highly relevant to the risks proposed in humans associated with mitochondrial replacement. There are now multiple reports of the health status of the offspring born after mitochondrial replacement, and all have shown no difference between these offspring and controls [6], [7], [15]. As highlighted in the reports, the macaques used for these experiments were not, as suggested by the authors of the recent commentary [10], highly genetically related, but some were from divergent subspecies with extensive differences in the rhesus macaque genome [6]. Thus, the experiments using the animal model closest to man have not shown any adverse effects from mitochondrial transfer.

Some studies in laboratory mice have proposed a nuclear DNA–mitochondrial DNA interaction, but there are others that have reported no defect despite the use of very divergent genomes [16]–[18]. It is important to recognise that these studies, and those in invertebrates, have been performed on highly inbred species (often inbred over thousands of generations) and the relevance to human populations must be questioned. Most human populations are outbred with considerable mixing of the genome over recent generations. In these populations the mixing of alleles will inevitably dilute the effect of potentially harmful nuclear DNA-mitochondrial DNA interactions. There has never been any direct evidence of a “mismatch” between the two in humans—either on an evolutionary scale or in the context of disease. This is even the case for couples from divergent haplogroups, where potential nuclear-mitochondrial mismatches are at their most extreme. Thus, from the mitochondrial DNA perspective, any mitochondrial transfer experiment is just recapitulating what is happening every day all around the world—and without any known adverse effects.

Whilst we accept that any new technique is associated with risk, we think the lack of any reliable evidence of mitochondrial-nuclear interaction as a cause of disease in human outbred populations provides the necessary reassurance to proceed. The recent studies in macaques after mitochondrial replacement are also supportive that the possible harmful interactions are unlikely to occur in man [6], [7]. Human preimplantation embryos and embryonic stem cells generated with “unmatched” mtDNA replacement demonstrated normal development and differentiation potential [7], [8]. As suggested by the HFEA [9], it is possible to match mitochondrial haplotype between the mother and the mitochondrial donor to avoid any concern, even though the evidence says it should not be needed. We do not believe this important development should be delayed—for families carrying mtDNA mutations, the clock is ticking, and the desire to have children free of mitochondrial DNA disease is entirely justified. Ultimately, we believe those that carry mitochondrial DNA mutations must be fully informed of the potential risks, and that they will decide which option to take.
