# Correction: The Challenges of Mitochondrial Replacement

**DOI:** 10.1371/journal.pgen.1004472

**Published:** 2014-06-02

**Authors:** The PLOS Genetics Staff

The Competing Interests section contains information that is incorrect. The correct Competing Interests statement is:

Oregon Health & Sciences University has a provisional patent application entitled "Method for mitochondrial DNA replacement in oocytes". SM is an inventor. PFC, LC, JBS, MH and DMT have declared that no competing interests exist.
